# Endoscopic cartilage myringoplasty with the removal of a small rim of the external auditory canal to repair marginal perforations

**DOI:** 10.1186/s40463-020-00408-7

**Published:** 2020-03-06

**Authors:** Zheng-cai Lou

**Affiliations:** Department of Otorhinolaryngology, Yiwu central Hospital, 699 jiangdong road, Yiwu city, 322000 Zhejiang provice China

**Keywords:** Tympanic membrane perforation, Endoscopy, Cartilage myringoplasty, Tympanomeatal flap, Lateralization

## Abstract

**Objective:**

To evaluate the graft success rate and postoperative hearing gain for marginal perforations using endoscopic cartilage myringoplasty with the removal of a small rim of the external auditory canal (EAC).

**Study design:**

Prospective case series.

**Materials and methods:**

We performed a prospective study in 41 patients with marginal perforations who underwent endoscopic cartilage myringoplasty with the removal of a small rim of EAC. Patients were followed up for 6 months.

**Results:**

Of the 41 patients with unilateral marginal perforation included in this study, the graft success rate was 100% (41/41). The mean ABG improved from 11.31 ± 9.71 dB preoperatively to 7.31 ± 2.32 dB postoperatively for small-and medium-sized perforations (*P* = 0.13); the mean ABG improved from 21.46 ± 8.39 dB preoperatively to 9.84 ± 2.41 dB postoperatively for large perforations (*P* < 0.05); the mean ABG improved from 28.79 ± 6.74 dB preoperatively to 10.13 ± 3.56 dB postoperatively for subtotal and total perforations (*P* < 0.05). There were no cases of graft lateralization or significant blunting or atelectasis or graft adhesions. Three patients developed postoperative otorrhoea and five patients had mild myringitis.

**Conclusions:**

Endoscopic cartilage myringoplasty with the removal of a small rim of the EAC is simple and feasible, showing a high graft success rate and minimal complications for repairing marginal perforations.

## Introduction

Tympanoplasty is the basic surgical process for treating chronic tympanic membrane (TM) perforations, and temporalis fascia is the most common graft material. However, underlay fascia graft tympanoplasty is challenging for marginal perforations because of a lack of residual TM, the fascia graft may fall away, resulting in reperforation [1]. Overlay tympanoplasty has a high success rate and has been particularly effective for large, anterior perforations. The primary disadvantages of this technique include increased technical demands of surgery and postoperative blunting or lateralization of the TM graft. Extensive disruption of the normal tissue relationships required in this procedure may lead to delayed healing or long-standing granular myringitis [2].

A variety of surgical techniques have been developed to increase the surgical success of treating marginal perforations, including sandwich graft tympanoplasty [3], over-under tympanoplasty [4], mediolateral graft tympanoplasty [5], the “window shade” technique [6], “hammock” tympanoplasty [7], loop underlay tympanoplasty [8], and anterior interlay myringoplasty [9]. However, tympanomeatal flap elevation (TFE) is a crucial step in these surgical techniques.The amount of tympanomeatal flap elevated varies depending on the location and diameter of the perforation. Recent some authors reported endoscopic tympanoplasty without TFE in the central perforations [10, 11] and with limited TFE in large marginal perforations [12–14]. TFE is technically difficult to integrally detach the tympanomeatal flap and form the tunnel because of the bleeding skin of the external auditory canal (EAC) and one-handed operation in endoscopic technique. Although butterfly cartilage myringoplasty does not require TFE for most TM Perforations, medial canal wall skin should be elevated to expose the bony annulus for large marginal perforations [15, 16]. This study explored endoscopic cartilage myringoplasty with the removal of a small rim of the EAC to repair marginal perforations.

## Materials and methods

### Ethical considerations

The study protocol was reviewed and approved by the Institutional Ethical Review Board of Yiwu Central Hospital in Yiwu, Zhejiang, China. Informed consent was obtained from all participants.

### Materials

This was a prospective case series study performed from August 2016 to December 2017. The inclusion criteria were marginal perforations with mucosal chronic otitis media (COM), no suspicion of an ossicular chain defect, conductive hearing loss no greater than 40 dB in any frequency, and dry ears for at least 2 months prior to surgery. The exclusion criteria included ossicular chain abnormalities, suspected cholesteatoma, and the presence of fungal otitis externa. Preoperative temporal bone CT/MRI was performed to exclude ossicular chain abnormalities and middle ear cholesteatoma.Perforation sizes were classified as subtotal and total (involving more than 75% of the eardrum area), large (involving more than 50% of the eardrum area), medium (involving 25–50% of the eardrum area), or small (involving less than 25% of the eardrum area) [17]. Perforation positions were classified as anterior, central, or posterior with respect to the handle of the malleus [18]. We defined the operative time as the duration from the start of fresh perforation edge to the end of wound dressing. The pure-tone average (PTA) was calculated as the mean of the pure-tone hearing thresholds at 500, 1000, 2000, and 3000 Hz. The air–bone gap (ABG) was determined as the mean of the differences between the air conduction thresholds and the bone conduction thresholds at 500,1000, 2000, and 3000 Hz before operation and at 6 months postoperatively.

### Surgical techniques

During endoscopic cartilage myringoplasty, patients were placed in the supine position with the head 30° up and oriented toward the opposite side with the video equipment placed on the opposite side of the surgeon.

**Fig. 1:**
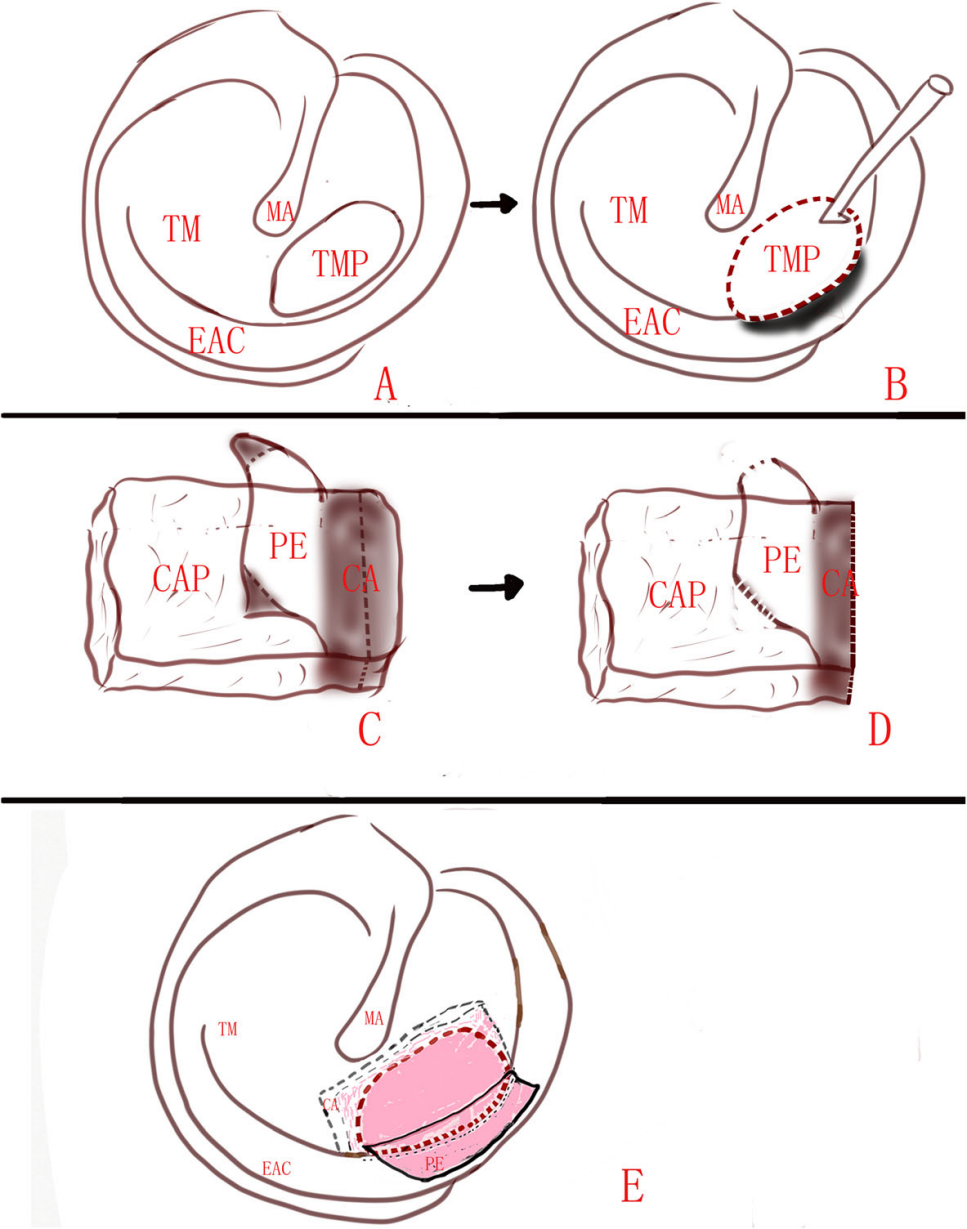
Diagram of the marginal perforations not involving the malleus. Tympanic membrane perforation (**a**); the perforation edges were refreshed and annulus was de-epithelialized, a small rim of EAC was removed (**b**); the perichondrium was listed off of the cartilage over 2 mm on one end (**c**); the uncover cartilage was partially removed (**d**); the cartilage covered by perichondrium was placed medial to the remnant TM, the free perichondrium was placed lateral to the annulus and exposed EAC (**e**)

**Fig. 2:**
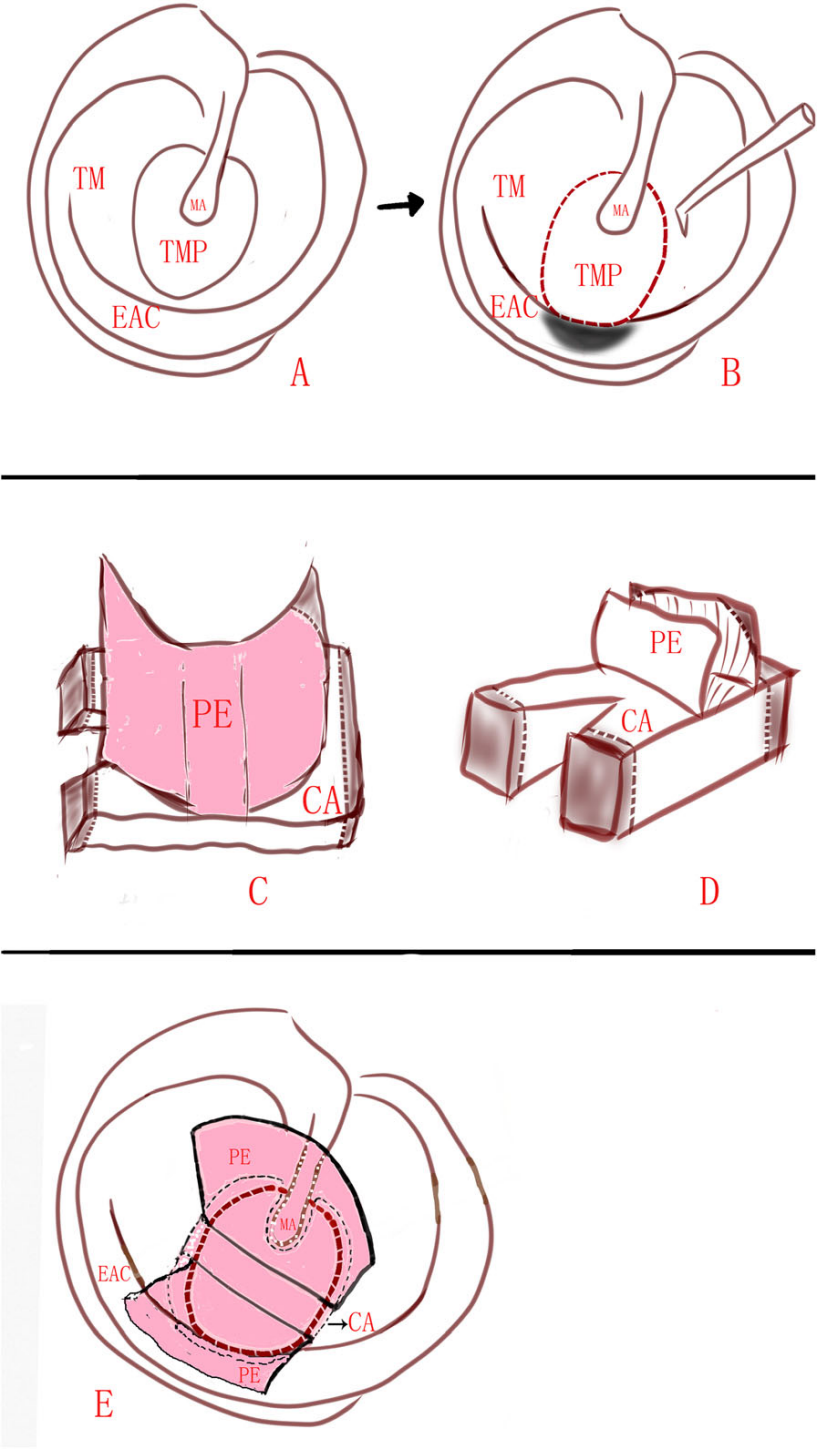
Diagram of the marginal perforations involving the malleus. Tympanic membrane perforation (**a**); the perforation edges were refreshed and annulus was de-epithelialized, a small rim of EAC was removed (**b**); the lateral perichondrium was lifted off of the superior and inferior end, a notch was made for the malleus (**c** and **d**); the cartilage graft was placed medial to the remnant TM and the annulus, a notch of cartilage was clipped into the malleus, the free perichondrium was placed lateral to the malleus, the annulus and exposed EAC (**e**). TM: tympanic membrane; TMP: tympanic membrane perforation; EAC: external auditory canal; MA: malleus handle; PE: perichondrium; CA: cartilage; CAP: cartilage with perichondrium. The black shadow region indicates the excision of a small rim of EAC. Red dotted line indicates the fresh perforation edges. The section E: Black hidden line indicated the underlay cartilage. Pink shadow indicates the overlay perichondrium

The perforation edges were visualized and refreshed under a 0° rigid endoscope, the annulus without remnant TM was de-epithelialized. If the perforation involves the malleus, the epithelium was removed from the distal malleus handle. Then, the removal of a small rim of corresponding EAC skin was performed to expose the EAC bone at least 2 mm wider (Figs. 1 and 2).

The graft composed of cartilage and perichondrium on one side was harvested from the ipsilateral tragus.The cartilage with perichondrium was fashioned based on the size of the perforation and the situation of the malleus. Under endoscopy, the middle ear was tightly packed with biodegradable Nasopore soaked in antibiotic ointment to the level of the perforation.

For marginal perforations not involving the malleus, the perichondrium was listed off of the cartilage over 2 mm on one end, edge of the graft and left attached to the cartilage on the other end. The uncover cartilage was partially removed and placed medial to the annulus, the cartilage covered by perichondrium was placed medial to the remnant TM in an underlay manner, however, the free perichondrium was elevated and placed lateral to the annulus and exposed EAC in an overlay manner (Fig. 1).

For marginal perforations involving the malleus, the lateral perichondrium was lifted off of the superior and inferior end. The cartilage notch was made for the malleus. Then, the cartilage graft was pushed through the perforation and placed medial to the remnant TM and the annulus in an underlay manner, the cartilage notch was accommodated the malleus. However, the free perichondrium of the superior and inferior end was placed lateral to the malleus, the annulus and exposed EAC in an overlay manner (Fig. 2).

**Fig. 3:**
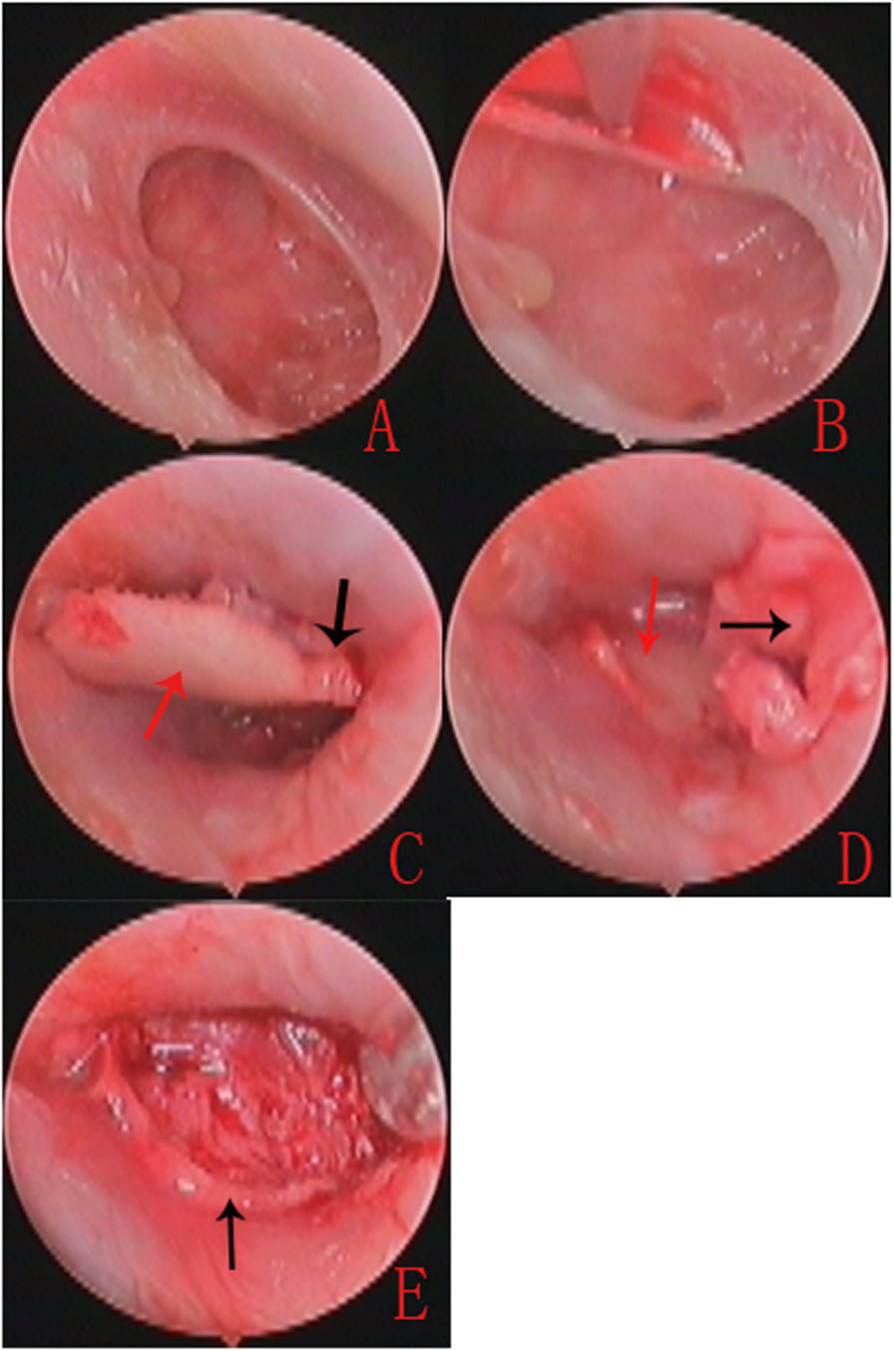
The operative photos of right large TM perforation in a 32-year-old female patient. Preoperative total TMP (**a**). The perforation edges were refreshed and annulus was de-epithelialized (**b**). Cartilage graft was pushed into the EAC and middle ear; the cartilage notch was made for the malleus (**c**). The cartilage graft was placed medial to the annulus and remanent TM (**d**). The free perichondrium graft was placed lateral to the malleus, the annulus and exposed EAC (**e**).Black arrows indicates the perichondrium, red arrows indicates the cartilage and notch

For large, subtotal and total perforations, the lateral perichondrium was lifted off all around the end and the pedicle attached the centre of the cartilage. The cartilage notch was made for the malleus. Then, the cartilage graft was placed medial to the annulus in an underlay manner, the free perichondrium was placed lateral to the malleus, the annulus and exposed EAC in an overlay manner (Fig. 3).

The EAC was first packed with Nasopore, followed by gauze soaked in antibiotic ointment, until the tragus incision was reached. The tragus incision was not sutured but pressured by gauze with antibiotic ointmen, and a small dressing was applied to cover the auricle.

### Postoperative follow-up

All patients were given a course of antibiotic (amoxicillin) postoperatively to prevent infection. Packing gauze soaked in antibiotic ointment for the EAC was removed 14 days postoperatively and biodegradable Nasopore fragments were aspirated from the EAC at 4 weeks postoperatively; this allowed the graft to be visualized. All patients were followed up in the ENT outpatient department at 2 weeks, 1 month, 3 months, and 6 months postoperatively.

Endoscopic otological examinations were performed. The primary outcome was the graft success rate at 6 months postoperatively. Audiological testing was conducted 6 months after surgery. Each patient had undergone a preoperative audiological evaluation, including determination of the pure tone air and bone thresholds.

### Statistical analyses

Statistical analyses were performed using SPSS version 20 (SPSS Inc., IBM Company, Chicago, IL, USA). The data are expressed as the mean (standard deviation [SD]) and percentage (%). Differences between preoperative and postoperative air–bone gaps were analysed using Wilcoxon’s signed-rank test. A *P*-value <0.05 was considered significant.

## Results

### Demographic data

The study population consisted of 41 patients with unilateral marginal perforation with COM (27 females and 14 males; average age 52.6 ± 13.7 years). Overall, 22 patients had left side involvement and 19 had right side involvement, and the mean duration of perforation was 21.3 ± 11.6 years. Perforations were small in 2 (4.9%) patients, medium in 7 (17.1%) patients, large in 26 (63.4%) patients, and subtotal and total in 6 (14.6%) patients. The perforation position was anterior in 22 (53.7%) patients, subtotal and total in 6 (14.6%) patients, and posterior in 13 (31.7%) patients. The mean operation time was 37.2 ± 11.9 min among the 41 patients.

### Graft take rate and hearing gain

**Fig. 4:**
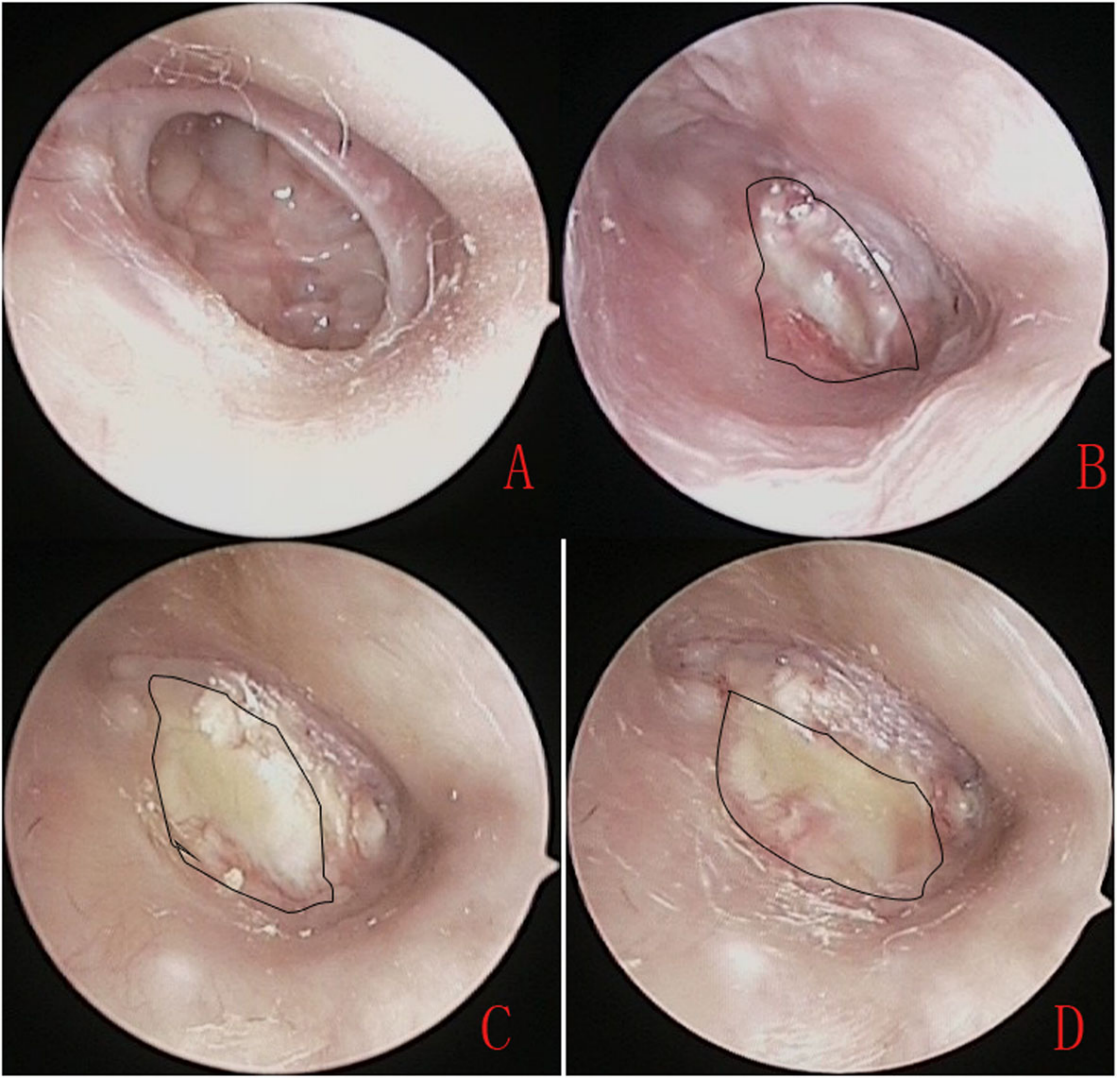
Photographs showing the perforation before surgery (**a**), and at 3 weeks (**b**), 5 weeks (**c**), and 6 weeks (**d**) post-surgery. Please note, this is the same patient as in Fig. 3. The irregular curve indicates the overlay perichondrium area

The tragal incision was closed completely in all patients. At 6 months, the graft success rate was 100% (41/41) and no residual perforation was seen (Fig. 4). The mean ABG improved from 11.31 ± 9.71 dB preoperatively to 7.31 ± 2.32 dB postoperatively for small-and medium-sized perforations (*P* = 0.13); the mean ABG improved from 21.46 ± 8.39 dB preoperatively to 9.84 ± 2.41 dB postoperatively for large perforations (*P* < 0.05, Wilcoxon’s signed-rank test); the mean ABG improved from 28.79 ± 6.74 dB preoperatively to 10.13 ± 3.56 dB postoperatively for subtotal and total perforations (*P* < 0.05, Wilcoxon’s signed-rank test). For the individual ABG closure percentages of perforation size,100% (9/9) had ABG closure within 10 dB in the small- and medium sized perforations; 80.8% (21/26) had ABG closure within 10 dB and 19.2% (5/26) had closure within 20 dB in the large perforations; 50.0%(3/6) had ABG closure within 10 dB,33.3% (2/6) had closure within 20 dB and 16.7% (1/6) had closure within 30 dB for the subtotal and total perforations.

### Complications

There were no complications, such as iatrogenic sensorineural hearing loss, altered taste, facial nerve palsy, vertigo, or tinnitus, during the follow-up period. There were no cases of graft lateralization, significant blunting, graft atelectasis, or graft adhesions, or effusion. Three patients developed postoperative otorrhoea, which resolved after they received otic and oral antibiotic treatments. Five (12.2%) patients had mild myringitis, which resolved after silver nitrate cauterization and oral antibiotic treatment. No any intra-tympanic cholesteatoma or keratin pearls was noted during the follow-up period.

## Discussion

Usually, the most challenging TM perforations for myringoplasty are the marginal perforations because of access difficulty and lack of support to the graft. TFE and creation of a tunnel are essential to improve graft stability in most cases [19–21]. In this study, however, we applied endoscopic cartilage myringoplasty with the removal of a small rim of the EAC to repair the marginal perforations. The graft success rate was 100% (41/41) and no residual perforation was seen at 6 months in this study.

Using this technique, a small rim of the EAC was removed while the integrity of the annulus was preserved. The cartilage graft was placed medial to the remnant TM and annulus to completely close the perforation using the underlay technique, while the free perichondrium attached the cartilage was placed lateral to the annulus and exposed EAC using an overlay technique to strengthen the perforation. The pedicle of the perichondrium attached the remnant TM and cartilage to ensure cartilage composite graft survival. The free perichondrium was placed on the exposed surface of the EAC, which was similar to EAC skin graft. Ghanem et al. reported a split-thickness skin graft in 69.6% of patients with large perforations using butterfly cartilage graft inlay tympanoplasty with a skin graft survival rate of 100% [22]. Only a small rim of EAC skin was removed, and the perichondrium overlay the EAC gradually became epithelialized through the remaining EAC skin. Previous studies have shown that the use of a large perichondrial flap in contact with the vascular strip and undersurface of the TM preserves cartilage viability [23], possibly through the low metabolic rate in the central part of the cartilage where there is minimal activity resulting in the cartilage remaining viable for a longer time, while the greater activity in the periphery may lead to early proliferation of blood vessels [23–26]. The proliferation of blood vessels further provides nutrition for the EAC perichondrium. None of any minor touchups was necessary during follow up period to enhance healing in this study.

The difference between the preoperative and postoperative ABG was significant except for the small- and medium sized perforations in this study. As larger perforation are far more likely to have larger hearing deficits than smaller prior to surgery. Previous studies have shown that despite cartilage graft stiffness, hearing does not appear to be negatively impacted by such grafts [27–29]. In addition, graft lateralization and significant blunting were not observed during follow-up in this study. The biodegradable Nasopore was removed 4 weeks after the procedure, which played a role in fixation of the perichondrium graft, thereby avoiding graft lateralization. No Nasopore-related complications, such as adhesions and effusion, were also observed. A previous study demonstrated that NasoPore, a biodegradable synthetic polyurethane foam, was a safe packing material for applications within the middle ear cavity, with only a mild inflammatory response in the middles ear mucosa, leading to fewer fibrosis and adhesions within the middle ear compared with Gelfoam-packing [30, 31].

However, five (12.2%) patients developed postoperative myringitis in this study. This high rate may have been associated with the exposed perichondrium on the surface of the EAC. Nevertheless, all cases of myringitis resolved after silver nitrate cauterization and oral antibiotic treatment. No any intra-tympanic cholesteatoma or keratin pearls was noted during the follow-up period in this study. The present technique was similar to previous study [32]. Ahmed S et al. performed Chondroperichondrial clip myringoplasty to repair small to medium-sized perforations [32]. In their technique, the excision of the epithelial layer of the TM remnant wasn’ t made. The cartilage component of the graft was engaged the perforation edge, while the overlying perichondrium was spread out over the TM remnant. They didn’t report intra-tympanic cholesteatoma or keratin pearls in the follow-up period which ranged from 12 to 26 months (mean 13 months).

The limitations of this study included the small sample size, short follow-up time (only 6 months), and no randomized controlled trial. It was unclear whether patients developed middle ear cholesteatoma over the long term. In addition, this study was not a randomized controlled trial. Postoperative CT/MRI should be performed to further monitor cholesteatoma formation in future.

## Conclusion

Endoscopic cartilage myringoplasty with the removal of a small rim of the EAC is simple and feasible, showing a high graft success rate and minimal complications for repairing marginal perforations.

## Data Availability

The datasets supporting the conclusions of this article are included within the article.

## Abbreviations

COM: Chronic otitis media
CT: Computed tomography
EAC: External auditory canal
TFE: Tympanomeatal flap elevation
TM: Tympanic membrane
